# Gametocidal genes: biological mechanisms owing to hybrid dysgenesis in crop breeding, challenges and innovation

**DOI:** 10.1093/aob/mcaf226

**Published:** 2025-09-22

**Authors:** Nicola Walter, Ian King, Julie King, Surbhi Grewal

**Affiliations:** Nottingham Wheat Research Centre, University of Nottingham, Sutton Bonington Campus, Loughborough LE125RD, UK; Nottingham Wheat Research Centre, University of Nottingham, Sutton Bonington Campus, Loughborough LE125RD, UK; Nottingham Wheat Research Centre, University of Nottingham, Sutton Bonington Campus, Loughborough LE125RD, UK; Nottingham Wheat Research Centre, University of Nottingham, Sutton Bonington Campus, Loughborough LE125RD, UK

**Keywords:** Gametocidal, cuckoo chromosomes, *Aegilops* spp, *Aegilops sharonensis*, gametes, hybrid sterility, wild relatives, *Triticum aestivum*, *Nicotiana* spp, *Oryza* spp, pollen killer, sterility

## Abstract

**Background:**

Climate change and population growth are major threats to global food security. Many cultivated crops remain vulnerable due to reduced genetic variation. Wild relatives and diverse accessions of crop species are being used to reintroduce diversity into their genomes to help contend with these issues. However, in some species, notably *Triticum aestivum*, *Oryza* spp., *Solanum lycopersicum*, *Zea mays* and *Nicotiana* spp., *Arabidopsis thaliana* and their wild relatives, gamete-killing genes may be responsible for the occurrence of hybrid dysgenesis through the targeting of reproductive cells that do not contain the gene.

**Scope:**

This article explores gametocidal genes, ‘pollen killers’ or ‘gamete killers’, and toxin–antidote systems that result in sterility, alongside potential biological mechanisms. Gametocidal genes from wheat wild relatives significantly impact breeding programmes: wild relatives may contain useful germplasm but also gametocidal genes resulting in disastrous effects, including yield reductions. Due to their preferential transmission, gametocidal genes are extremely difficult to remove, therefore gene characterization is necessary. Hybrid sterility loci in *Oryza* spp. have been addressed, highlighting those that function similarly to gametocidal genes. We collate recent evidence to appraise the merit of biological mechanism hypotheses and suggest how recent innovations may improve characterization. Additionally, the challenges that they contribute to breeding programmes and subsequent successes are highlighted. In light of genetic innovation, we suggest contexts where a revival of using gametocidal genes may be beneficial, alongside novel techniques for research.

**Conclusions:**

Past research has identified unique characteristics of gametocidal genes, leading to theories such as the dual-mechanism and restriction-modification models to explain the mechanisms. However, recent research suggests that complex genetic factors such as transposable elements and epigenetics may account for the phenomenon. Future work towards mapping these genes is hopeful: innovations in sequencing, bioinformatics and genomic data have improved the ability to precisely identify the elusive gametocidal genes.

## INTRODUCTION

Eliminating hunger and malnutrition remains high on the FAO’s list of sustainable development goals. Furthermore, growing populations have caused concern for achieving future global food security (FAO *et al.*, 2021). This is alongside the continuing threat of climate change forcing more difficult conditions on farmers for food production. Pest and disease damage is set to become worse due to a hotter, more humid climate in many current high-cropping areas, alongside extreme weather events. A major line of research to mitigate these challenges has been future-proofing crops. It is of the highest priority to establish high-yielding varieties with increased tolerance to heat, drought, flooding and salinity alongside the plethora of pests and diseases that currently and will threaten our crops to achieve global food security in the future.

Many crop species have been, whether unconsciously or not, selectively bred to improve yields, ease of harvesting or suitability for the field. In doing so, while genes for visible traits, such as large fruit size, have been retained, many genes for traits that are not necessarily visible, such as disease resistance, have been lost, leaving crops vulnerable and with limited genetic diversity. These genetic bottlenecks hinder our ability to develop new, beneficial traits. A large line of inquiry has been the use of wild relatives of crop species to reintroduce genetic diversity back into cultivated crops. Due to their homoeologous nature, chromosomes are often able to recombine, bringing in new diversity that can be tested for novel traits. For example, germplasm originating from a variety of genera from the Triticeae tribe has been successfully introduced into *Triticum* (including *T. aestivum*, bread wheat), *Aegilops*, *Thinopyrum*, *Hordeum* and *Secale* (Molnár-Láng *et al.*, 2015; King *et al.*, 2017, 2018, 2022; Millet *et al.*, 2017; Grewal *et al.*, 2018, 2020*b*; Kishii, 2019; Fellers *et al.*, 2020; Steed *et al.*, 2022). Consequently, during phenotypic analysis, wheat–wild relative introgression lines have been observed to present significant traits that could further improve elite wheat cultivars, such as resistance to fusarium head blight from *Triticum timopheevii* (Steed *et al.*, 2022). Resistances to wheat rust diseases have been identified in the wild relative and wheat-wild relative introgression lines with *Aegilops mutica* (Fellers *et al.*, 2020), *Ae. speltoides* (Li *et al.*, 2023; Ragini *et al.*, 2024), *Ae. longissima* (Xu *et al.*, 2024) and *Ae. sharonensis* (Marais *et al.*, 2006; Millet *et al.*, 2017; Sharma *et al.*, 2024). The last of these has additionally been found to have resistances to powdery mildew (Olivera *et al.*, 2007; Wang *et al.*, 2021).

However, hybrid dysgenesis or hybrid necrosis is often observed in some intergeneric and interspecific hybrids, where plants have lost vigour and normal growth and development, small, shrivelled seeds, and often exhibit full or part sterility. In some instances, gametocidal genes may be responsible for the occurrence of hybrid dysgenesis, and have been noted under varying terminology in wheat (Loegering and Sears, 1963; Endo, 1988*a*), *Aegilops* spp. (Endo, 1990, 2007, 2015; Tsujimoto, 2005; Molnár-Láng *et al.*, 2015; Said *et al.*, 2024), *Oryza* spp. (Sano *et al.*, 1979; Sano, 1990; Long *et al.*, 2008; Xie *et al.*, 2017*a*, *b*; You *et al.*, 2023; Wang *et al.*, 2023*b*; Myint *et al.*, 2024), *Solanum lycopersicum* (Rick, 1966), *Nicotiana* spp. (Cameron and Moav, 1957), *Zea mays* (Xue *et al.*, 2019), *Arabidopsis thaliana* (Simon *et al.*, 2016) and their wild relatives. These gamete-killing elements are distinct from general hybrid necrosis, hybrid dysgenesis or incompatibility of gametes, due to the unusual phenomenon whereby gametes that do not contain a copy of the gene are selectively targeted. Through this, they ensure their preferential transmission to offspring. Without removal of the gametocidal trait, wild relative introgression lines will remain agronomically unsuitable and cannot be used to improve elite crop varieties.

## INITIAL DISCOVERIES OF GAMETOCIDAL GENES

In essence, gametocidal genes are selfish elements that ensure their transmission to offspring through the induction of double-stranded chromosomal breakages in gametes that lack them. Consequently, they are a type of segregation distorter and reject Mendelian genetics in favour of themselves. The first observation of gametocidal action was likely by Cameron and Moav (1957), when the addition of a *Nicotiana plumbaginifolia* chromosome to *N. tabacum* caused pollen abortion of gametophytes in which the *N. plumbaginifolia* chromosome was not present. Gametocidal action occurred immediately following the release of the microspores from the tetrad, yet female gametes appeared unaffected. Consequently, this was entitled the ‘pollen killer’ locus, and nearly always conferred preferential transmission of the donor *N. plumbaginifolia* chromosome. As megagametophytes became separated after nuclear division, it was thought that the effect only occurred when nuclei share common cytoplasm.

In 1960, Luig observed the similar phenomenon of differential transmission of gametes in wheat, writing a short letter to *Nature* journal (Luig, 1960). Subsequently, in 1962 Nyquist discussed the mystery surrounding the preferential transmission of several *Triticum timopheevii* segments in hybrids of the wild relative with wheat (Nyquist, 1962). Neither Luig nor Nyquist could fully explain the results. However, a year later, Loegering and Sears (1963) reported substantially on a ‘pollen-killing’ gene in wheat. The authors observed a similar pollen killer gene in ‘Timstein 6B’, a wheat cultivar ‘Timstein’ with its Chr. 6B replaced with Chr. 6B of cultivar ‘Chinese Spring’ (CS). The authors believed the gene to be carried by CS Chr. 6B, which caused lethality in gametes containing one or two copies of ‘Timstein’ Chr. 6B. They related this to the findings of Cameron and Moav (1957) in that the abortive effect only appeared to occur on male gametes but not female. However, the work implied that the gametocidal gene became active following the second division of meiosis, which ceased the development of pollen. Though no papers were cited, the authors suggested the discovery of this pollen-killing gene explained variable results in previous breeding programmes that utilized the ‘Timstein’ cultivar.

In 1966, a ‘gamete eliminator’ was discovered, of which the notable change in title reflected how the element affected not just male gametes, but both male and female gametes (Rick, 1966). In tomatoes, hybrids between an ‘unfruitful’ plant and other cultivars indicated that this factor was recessive in *F*_1_ generations. However, in *F*_2_ generations some cultivars segregated abnormally and aborted both pollen and ovules. It was deduced that the gamete eliminator lay within heterochromatin close to the centromere on Chr. 4 (Rick, 1971).

The term ‘gametocidal’ was coined in 1975 by Maan, who concluded that gametocidal action was occurring, caused by an element carried on *Aegilops longissima* and *Ae. sharonensis* chromosomes, rendering infertile any gametes not carrying the chromosome. This coincided with a paper by Endo and Tsunewaki (1975), who came across the suspected gametocidal action when studying cytoplasmic relationships between *Aegilops triuncialis*, *Ae. umbellulata* and *Ae. caudata*. They found that *Ae. umbellulata* and *Ae. caudata* were the source of an extra chromosome in the alloplasmic substitution lines, with *Ae. caudata* carrying the gametocidal effect. In later research, Endo (1978) further investigated the *Ae. triuncialis* chromosome carrying the gametocidal element, concluding that it was homoeologous to wheat group 3 chromosomes.

Endo (1990) extensively reviewed the known gametocidal chromosomes in *Aegilops* spp., noting the homoeologous relationship of wheat and *Aegilops* spp. chromosomes, alongside the chromosomal location of their subsequent translocations and mode of action. Endo subsequently proposed grouping the gametocidal genes according to their type of action, corresponding to their homoeologous groups 2, 3 and 4. In this review Endo reported 12 gametocidal chromosomes in six *Aegilops* species.

Through intergeneric hybridization of *T. aestivum* and *Aegilops* spp. followed by backcrossing, gametocidal genes have been unintentionally introduced into common wheat. In an updated review by Endo in 2007, the number of known gametocidal chromosomes had increased to 20 within eight species. Endo reviewed the gametocidal wheat–*Aegilops* spp. translocation lines, and used C-banding to demonstrate the gametocidal segment locations on the chromosomes (Endo, 2007). At the time, gametocidal genes were attributed to the C, S and M genomes (Endo, 2007). Said *et al.* (2024) recently reported 12 gametocidal genes in nine species, a lower figure than stated by Endo (2007). This may be attributed to improved technological advances that have linked genes that were previously reported as independent.

From 1975 and the publication by Endo and Tsunewaki, gametocidal genes within different genera, for example wheat and tomatoes, appeared to be no longer considered as potentially related, with reviews on the genes being grouped by genus (Endo and Tsunewaki, 1975). While similarities between gametocidal elements of different clades, even kingdoms (such as P*-*elements in *Drosophila* spp.; Ronsseray *et al.*, 1998), have been frequently noted, there has been little more insight. In the plant kingdom, gametocidal genes have been discovered in wheat (Loegering and Sears, 1963; Endo, 1988*a*) and its wild relatives (Endo, 1990, 2007, 2015; Tsujimoto, 2005; Said *et al.*, 2024), rice (Sano *et al.*, 1979; Sano, 1990; Long *et al.*, 2008; Xie *et al.*, 2017*a*, *b*; You *et al.*, 2023; Wang *et al.*, 2023*b*; Myint *et al.*, 2024), tomatoes (Rick, 1966), tobacco wild relatives (Cameron and Moav, 1957), maize (Xue *et al.*, 2019), *Arabidopsis thaliana* (Simon *et al.*, 2016) and many wild relatives of these plants, though there has been more research on *Aegilops* spp. and *Oryza* spp. than on other species.

## HYPOTHESES FOR GAMETOCIDAL ACTION

The exceptional feature of gametocidal elements, distinguishing them from other types of gamete abortion and reproductive isolation, is the lethality of gametes that do not contain the locus. Sterility of gametes is not unusual, with many genetic mutations or environmental conditions leading to gamete abortion (Snider and Oosterhuis, 2011; Khan *et al.*, 2023). However, gametocidal elements retain an ability to protect gametes that also contain the gene. Despite an awareness of gametocidal genes for decades, the biological mechanism is yet to be deciphered. However, early research has helped to develop theoretical models.

### Fundamentals of gametocidal action in *Aegilops* spp.

Finch *et al.* (1984) were among the first to undertake technical experiments to start identifying the biological mechanisms of gametocidal genes in wheat wild relatives. The authors observed both microspores and megaspores at varying stages of development in hemizygous wheat – *Ae. sharonensis* monosomic addition lines (2*n* = 42 wheat + 1 *Ae. sharonensis*, purported to be Chr. 4S^sh^). Both male and female meiosis appeared normal, but a large proportion of embryo sacs and some pollen grains were visibly abnormal at anthesis. They deduced that gametocidal action arose between these two periods. Further investigation revealed that at the first mitosis around half of both microspores and megaspores contained unusual chromosomal fragments, and it was inferred that gametocidal action arose just prior to this event in gamete development.

Nasuda *et al.* (1998) were further able to refine the timings of gametocidal action in male gametophytes. Using a wheat–*Ae. sharonensis* translocation line with the Chr. 4S^sh^ gametocidal element, and also wheat–*Ae. speltoides* (Chr. 2S) and wheat–*Ae. cylindrica* (Chr. 2C^c^L) addition lines, the authors cytologically observed microspores during meiosis and several stages of mitosis. Meiosis in all lines was considered cytologically normal. In homozygous lines, both first and second mitoses were also normal. However, it was clear that in hemizygous lines chromosomal fragments were observed, particularly in lines with *Ae. sharonensis* and *Ae. speltoides* gametocidal elements, corroborating previous findings of Finch *et al.* (1984). A higher frequency of normal mitosis arose in the second stage compared with the first, suggesting that cell cycle arrest had occurred as a result of the lethal gametocidal action. Through staging, the authors deciphered that the chromosomal fragmentation occurred during anaphase and telophase of both mitoses, specifically at the first post-meiotic interphase between G_1_ and S phase.

However, gametocidal action may occur at different times, not just between genera but also species. In a wheat line containing gametocidal Chr. 3C^t^ of *Ae. triuncialis*, Murata *et al.* (2018) observed chromosomal fragmentation distinctly before anaphase and telophase, in prometaphase and metaphase of the first pollen mitosis. This may suggest differences in evolutionary origin, particularly when considered alongside differences in strength.

While the biological mechanisms remained elusive, identification of the timing of gametocidal action provided a step change in the approach to further analyses. Furthermore, an entirely different stage of gametocidal action was identified. Endo (1990) concluded from the evidence of numerous studies that gametocidal action was also occurring in zygotes. King and Laurie (1993) confirmed this, using crosses between wheat cultivar CS, which was monosomic for Chr. 4B, and a Chr. 4B–4S^sh^ wheat substitution line. The authors cytologically observed chromosomal fragmentation in early embryo development, but only when the Chr. 4S^sh^ donor was the male parent, particularly 28–32 h post-fertilization. Additionally, regardless of parentage, chromosome aberrations occurred in the endosperm.

A key factor in gametocidal action is, as its name suggests, limited to gametogenesis, embryo and early endosperm development, and does not occur in somatic cells. To understand why, de Las Heras *et al.* (2001) attempted to induce chromosomal breakages in root tips of wheat cultivar CS with monosomic and disomic additions of Chr. 4S^sh^ of *Ae. sharonensis.* When treated with 5-azacytidine, a demethylation agent, root tip cells of plants with the Chr. 4S^sh^ additions resulted in chromosome fragmentation, similar to those perceived in early embryo and endosperm development (King and Laurie, 1993), pollen (Nasuda *et al.*, 1998) and both gametes (Finch *et al.*, 1984). This suggested that genomic imprinting was a major factor in preventing gametocidal action in somatic cells.

With this fundamental characterization of gametocidal action in *Aegilops* spp., models were devised to theorize what mechanisms may be occurring in this species. Here, the models are considered alongside the informing evidence and analysed against more recent studies.

### Dual-mechanism model

In 1956, upon discovery of a gametocidal element in an *N. plumbaginifolia* and *N. tabacum* hybrid, Cameron and Moav recognized the dual nature of the elusive element. The cells that did not contain the gametocidal locus were subject to the adverse effect, while cells that did contain the locus remained immune. The authors compared the antigenic theory with blood types, or the lethal agents produced by *Paramecium aurelia* (*P. aurelia* are ciliates, a group of alveolates characterized by the presence of cilia (Preer *et al.*, 1974); in some genotypes, *kappa*, symbiotic bacteria, are found in the cytoplasm, and can change to P particles that secrete paramecin, a poison that kills other strains of *P. aurelia* while they themselves remain immune).

In wheat, Endo (1990) also alluded to a dual function of gametocidal chromosomes, indicating that while they caused chromosomal aberrations in gametes that do not carry the chromosome, they also enabled protection of themselves against it. This was deduced from unpublished data suggesting that gametocidal action was dosage-dependent. Endo (1988*a*) observed that fewer abnormalities were observed in disomic addition hybrid lines than monosomic, which were again less than nullisomic addition lines. This highlighted that the higher the dosage of the gametocidal chromosomes in the addition lines, the more protection appears to be afforded to the gamete. Endo (1990) therefore discerned that a protective factor must occupy the gametocidal chromosome.

Since then, emerging evidence has supported this. In an experiment in which demethylated root tips of wheat lines containing a Chr. 4S^sh^ addition in various doses, upon treatment with 5-azacytidine, de Las Heras *et al.* (2001) noticed that more frequent aberrations occurred in those lines disomic for the gametocidal chromosome than in monosomic and nullisomic lines. Wang *et al.* (2017) further observed that differential methylation between addition lines correlated with the dosage of Chr. 3C^t^ of *Ae. triuncialis.* These experiments may suggest that methylation is a potential mechanism for the protective element of a gametocidal chromosome.

The most promising supportive evidence arose during the characterization of a mutant gametocidal line developed by Friebe *et al.* (2003). Friebe *et al.* produced images of the chromosomal breakage during male microgametogenesis in *Ae. sharonensis*, demonstrating that the breakages are induced in pollen cells lacking the gametocidal gene *Gc2.* In fluorescence *in situ* hybridization (FISH) images, the study demonstrated that in individuals hemizygous for *Gc2* (*Gc2*/−) non-*Gc2-*containing pollen cells were subject to these chromosomal aberrations. This corroborated the likelihood of a dual mechanism; one element, the ‘breaker’, caused aberrations in all chromosomes lacking *Gc2*, while a protective element, the ‘protector’, guarded the *Gc2*-carrier chromosomes from the breakage factor. Friebe *et al.* further tested this theory through a deletion study, using ethyl methanesulfonate (EMS) to induce mutations in the *F*_1_ generation of a cross between a T4B–4S^sh^ translocation line and CS. Plants were selected for 100 % seed set, which suggested a faulty breaker gene (only half the gametes would acquire the gametocidal gene in the *F*_1_ generation heterozygous for wild-type *Gc2*, resulting in 50 % gamete survival and therefore 50 % seed set). The progeny of *Gc2^mut^*/*Gc2^mut^* and *Gc2*/*Gc2* (offspring being *Gc2^mut^*/*Gc2*) underwent normal pollen mitosis and had fully fertile spikes. This suggested that *Gc2^mut^* has an intact protective factor, guarding it from the chromosomal breakage induced by *Gc2* and that the mutation is dominant.

### Suppressive elements

Suppressive genetic elements are thought to exist in several wheat cultivars. The hypothesis relates to the dual-mechanism theory, where this suppressor gene originates from a protector element to a corresponding breaker element that naturally developed a knockout mutation (Friebe *et al.*, 2003). Depending on the zygosity of the suppressive element, it may potentially be considered a protector element. A suppressor element may arise through a gametocidal gene in that species that has differentiated enough to lose its breaker action. However, the protective ability is still retained. For example, the breaker and protector elements were separated during crossing over and segregation during meiosis. Only further investigation to map suppressive elements and explore phylogeny, and cytological analysis of crosses between cultivars with suppressive abilities and gametocidal lines, may confirm the relatedness between suppressive and protector elements.

These suppressive elements have been found to inhibit gametocidal action, while seemingly not conferring gametocidal action themselves. Suppression of gametocidal action was described in a translocation line of Chr. 3C^t^ of *Ae. triuncialis* in the wheat cultivar ‘Norin 26’ background (Endo, 1978, 2007; Su *et al.*, 2013). Despite the gametocidal segment being present, the line was fully fertile. It was presumed that the suppressor gene was carried within the wheat cultivar. Tsujimoto and Tsunewaki (1985) located the suppressor gene on Chr. 3B in Norin 26 and designated it *Igc1* (meaning ‘first inhibitor of the Chr. 3C^t^ gametocidal gene’). Since the C-genome gametocidal genes exhibit homoeology to wheat group 3 chromosomes (Endo, 1990), Tsujimoto and Tsunewaki suspected a related origin. Furthermore, 65 different global cultivars (CS was not present) were tested for the suppressive action. It was found that all those carrying the *Igc1* locus were of Japanese origin, particularly southwest and central Japan. Given the findings of Tsujimoto and Tsunewaki, future studies may find useful an initial focus on cultivars deriving from this region.

An attempt to map *Igc1* found the location to be pericentromeric on Chr. B of Norin 26 (Yamano *et al.*, 2010). Yamano *et al.* explained that the central location of *Igc1* reduces the chance that the element that causes chromosomal breakage and the element that supresses the breakage will be separated during crossing over and segregation during meiosis. Since the location of the Chr. 3C^t^ gametocidal gene in *Ae. triuncialis* is yet to be mapped, the theory is still speculative. Arguably, the paper made the assumption that *Igc1* and the Chr. 3C^t^ gametocidal gene are related, which only added bias to this theory and the dual-mechanism theory (Endo, 1990). While it appears to support the dual-mechanism theory, there are numerous other hypotheses that also need to be considered and tested.

Gametocidal suppression has also been documented in CS. Endo (1988*b*) found that Chr. 2C^c^ of *Ae. cylindrica* induced severe gamete abortion and sterility in the cultivar ‘John Fife’ yet induced only chromosomal structural changes in the CS background. This could indicate, in CS, the presence of a suppressor element for group 2 gametocidal genes. The observation that *Igc1* was found on a homoeologous chromosome to the gametocidal gene (Tsujimoto and Tsunewaki, 1985) also supports a CS group 2 suppressor. Since the dosage of protective elements affects the degree of gametocidal action (Endo, 1988*a*), an initial investigation using monosomic and disomic CS lines for group 2 chromosomes may illuminate the presence of suppressors.

### Restriction-modification system

The dual-mechanism model has been a useful system to study gametocidal genes, though it remains ambiguous with regard to detail. In 2005, Tsujimoto suggested a system that appeared to build upon the dual-mechanism model, with considerations from systems outside of the plant kingdom. The author noted the similarity between gametocidal action and the restriction-modification system in some bacteria (Wilson and Murray, 1991). In this system, restriction enzymes are produced that cleave a specific site in the genome, unless the sites are methylated by the modification enzymes. Tsujimoto’s proposed model worked similarly; the gametocidal gene produces a restriction enzyme that cleaves specific sites in the genome, unless the sites are protected by DNA methylase, also produced by the gametocidal gene. In homozygous plants, all sites would be protected by methylation, whereas incomplete methylation, for example in gametes that do not contain the gene, would be subject to the restriction enzyme on these sites. While Tsujimoto (2005) cited the observations of chromosome breakages under hypomethylation treatment (de Las Heras *et al.*, 2001) to explain the theory, the experimental evidence was insufficient to support it.

In contrast to the dual-mechanism model (Cameron and Moav, 1957; Endo, 1990) and the restriction-modification system (Tsujimoto, 2005), some publications have briefly suggested that gametocidal action may be the result of multiple genes. For example, in attempts to remove the gametocidal gene, Marais *et al.* (2010) and Ragini *et al.* (2024) observed that fertility in progeny over generations continued to vary. While Ragini *et al.* claimed to have completely removed gametocidal action, Marais *et al.* considered that the gametocidal gene may have multiple elements, with more being removed with each successive backcross.

### Development of theories

The dual-mechanism theory, further developed with the restriction-modification system, with one gametocidal breaker element and a protector element, has become the commonplace model to understand gametocidal action. Despite advances in our understanding of genetics, little has been added to this decades-old theory. Furthermore, there are still significant gaps in knowledge and the lines of inquiry that have been pursued have been limited. The primary source of testing has been wheat wild relatives. Perhaps owing to their significance in breeding programmes and wheat’s role in global food security, a focus has been maintained on overcoming the issues associated with gametocidal genes. However, many plant species have demonstrated gametocidal action, or phenomena with significant similarities. Recent research has not considered the similarities and potential to identify key traits common to all.

The dual-mechanism models may have streamlined thinking towards gametocidal genes remaining distinct for each species. However, understanding these discrepancies may hold the key to deciphering many aspects of the mechanism. For instance, should commonalities of gametocidal action in rice or maize be identified, this may infer that gametocidal action is a cellular process, common to organisms across the plant kingdom under specific circumstances. Furthermore, occurrences of similar dysgeneses have been reported in the animal kingdom. Certainly, caution should be adopted when comparing such vastly different organisms, but there is still much to be considered when comparing basic cellular processes. Here, a wider approach has been adopted to consider literature beyond that of wheat wild relatives.

#### Hybrid dysgenesis resulting from transposable element activation

Hybrid dysgenesis is frequently caused by transposable elements (TEs). Transposable elements, also known as ‘jumping genes’, are highly repetitive, selfish elements that are able to mobilize around the genome using cut-and-paste mechanisms. Found in almost all organisms, both prokaryotic and eukaryotic, they accumulate in genomes over time through repetitive mobilization, explaining the significant proportion they may occupy. For example, the wheat genome is estimated to be made up of 85 % TE copies of 505 families (International Wheat Genome Sequencing Consortium (IWGSC) *et al.*, 2018*b*), 46.64 % of the genome in *Oryza sativa* (Ou *et al.*, 2019), 65.66 % in *Solanum lycopersicum* (Su *et al.*, 2021), 88.37 % *Zea mays* (Chen *et al.*, 2023), and 82.3 and 78.7 % in the *Ae. sharonensis* and *Ae. speltoides* genomes respectively (Avni *et al.*, 2022).

Transposable elements have had a significant influence on evolution. Organisms have evolved several strategies to maintain genomic stability through repressing expression of TEs alongside intercepting them post-transcription, while still benefitting from genetic variability through the occasional transposition event.

Most organisms have mechanisms that make hybridization between other genera or species difficult or impossible. For example, *Ph1* in wheat specifically ensures recombination between homologues, not homoeologues, which likely arose as a mechanism to contend with polyploid genomes (Martín *et al.*, 2017). Some genomes are so divergent that genes are no longer collinear and cannot naturally recombine. Additionally, many offspring of successful intergeneric or interspecific crosses are sterile, preventing further advances of the hybrid. For this reason, many organisms have evolved TE strategy artefacts, such as small interfering RNAs (siRNAs) that are specific only to their genome.

Hybrid dysgenesis affects offspring of crosses from different strains, species or genera and brings about genetic distortion. This can include chromosomal rearrangements, mutagenesis, increased recombination frequency, chromosome breakage, sterility and abortion of gametes. Most notably, it has been reported in *Drosophila* spp. owing to enhanced TE activity (Malone *et al.*, 2015). The most famous account of hybrid dysgenesis in *Drosophila* spp. is the P-M system, whereby autonomous TEs known as P-elements relocate their position within the genome of a germ-line cell (Kidwell *et al.*, 1977; Crow, 1984). There are two types of flies, P-strain and M-strain: P-strain flies possess the P-elements. When a male P-strain mates with a female M-strain, hybrid dysgenesis occurs whereby the P-element can transpose, or move, to different locations in the genome of germ-line cells, potentially becoming mutagenic if it lands within a gene (Muñoz-Lopez and Garcia-Perez, 2010). However, when a male M-strain mates with a female P-strain, hybrid dysgenesis does not occur. This is because there is a high concentration of P-element repressor molecules active in P-strain eggs that prevent the transcription of transposase, the enzyme needed for transposition.

Hybrid dysgenesis by TEs typically occurs when one parent carries active TEs that the other parent lacks. In the P-M system, alongside other documented dysgenesis occurrences, such as the I-R system (Bucheton *et al.*, 1984) and the *Penelope* transposon in *Drosophila virilis* (Lozovskaya *et al.*, 1990), dysgenesis occurs exclusively in the offspring from crosses where the male parent possesses TEs that are absent in the female parent.

While *Drosophila* species are by far the most prominent model of hybrid dysgenesis, plant species have also been known to exhibit the syndrome, also known as hybrid decay or hybrid necrosis. In *Arabidopsis*, differences in ploidy have often been to blame for inviability of offspring. However, Martienssen (2010) noted that in light of the finding that siRNAs can transfer from the vegetative nucleus to the sperm cell in developing pollen, enhanced TE activity may be the cause of dysgenesis symptoms. The A and T genomes of *Arabidopsis thaliana* and *Arabidopsis arenosa*, respectively, differ substantially in their TE sequences and centromeric repeats. It was suggested that when siRNAs produced in pollen do not match TEs in the central maternal cell and vice versa, it may result in enhanced TE activity.

Additionally, de la Luz Gutiérrez-Nava *et al.* (1998) found that a hybrid between Zapalote Chico, a Mexican maize landrace, and another maize germplasm resulted in hybrid dysgenesis. It occurred due to the activation of a mutator transposon during this cross. Furthermore, Xue *et al.* (2019) discussed the occurrence of transgenerational epigenetic hybrid decay in maize and its ancestral species teosinte. An *F*_1_ between maize and teosinte resulted in normal offspring, but subsequent backcrosses of this *F*_1_ with maize resulted in a sickly phenotype that increased in severity in further backcrosses. An increase in TE copy number was found in backcrossed populations, making it likely that the decay was due to activation of previously silenced TEs in the teosinte genome.

Rice has also been known to be affected by TE-induced hybrid dysgenesis. Nadir *et al.* (2019) suggest LTR retrotransposons may be the cause of hybrid weakness in intra-subspecific hybrids in japonica rice. Furthermore, sequence analysis of small RNAs of synthetic wheat, an artificially recreated hexaploid genome obtained through crossing tetraploid wheat *Triticum turgidum* ssp. durum cultivar ‘Langdon’ (genome BBAA) with *Aegilops tauschii* (genome DD), has also revealed that TE and siRNA changes may contribute to genome destabilization, in a similar way to hybrid dysgenesis (Kenan-Eichler *et al.*, 2011).

#### Commonalities of gametocidal genes, hybrid dysgenesis and transposable elements

There are undeniable similarities to the symptoms of gametocidal genes and TE-induced hybrid dysgenesis. The dual-mechanism model suggested by Endo (1990) built on research undertaken by Tsujimoto and Tsunewaki (1985), who originally suggested the P-M system in *Drosophila* spp. hybrids to explain gametocidal action. In hybrid dysgenesis induced by the P-M system, symptoms include chromosomal rearrangements, mutagenesis, increased recombination frequency, chromosome breakage, sterility and abortion of gametes. As noted, these are near-identical to the symptoms observed during gametocidal action in wheat wild relatives.

**Fig. 1.**
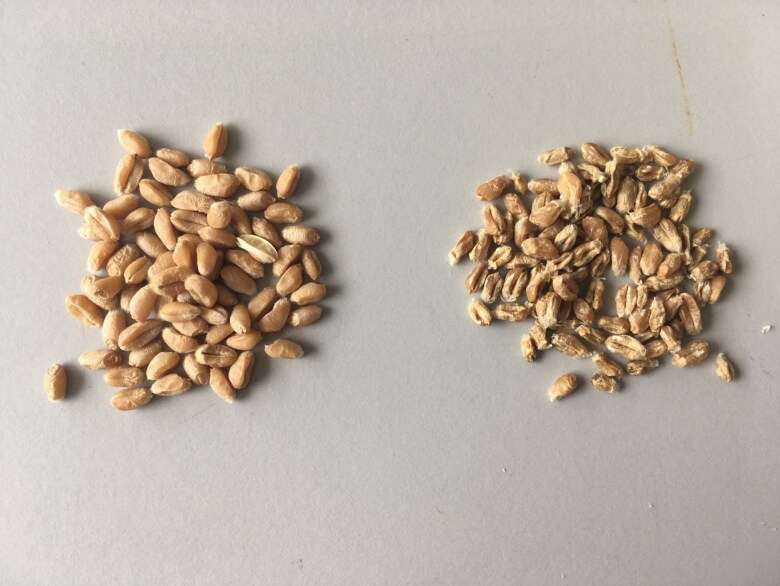
Side-by-side comparison of typical wheat cultivar ‘Chinese Spring’ seeds (left) and gametocidal introgression line T4B–4S^sh^ (right; Nasuda *et al.*, 1998). T4B–4S^sh^ contains a segment from wild relative *Aegilops sharonensis* on the tip of Chr. 4BL, in a ‘Chinese Spring’ background. Gametocidal lines are often associated with shrivelling of seeds (Tsujimoto and Tsunewaki, 1985).

Xue *et al.* (2019) commented on the transgenerational hybrid decay in *F*_1_ crosses between maize and teosinte. The *F*_1_ had a normal phenotype, though subsequent backcrosses resulted in an increasingly severe, sickly phenotype. This poses a great similarity to that of *Gc2*-containing wheat–wild relative introgression lines; the *F*_1_, with wheat and introgression line parents, has a normal phenotype, whereas a subsequent self-crossed or backcrossed generation has semi-sterility, shrivelled seeds and sickly phenotype (Fig. 1; Knight *et al.*, 2015).

Furthermore, the timing of gametocidal action has been narrowed down to the interphase prior to the first mitosis of microgametogenesis (Finch *et al.*, 1984) and also in embryos (King and Laurie, 1993). This corresponds to the timing of TE activation during hybrid dysgenesis, which occurs during gametogenesis and embryogenesis.

The similarity of the location of *Gc2* can also be considered. Ronsseray *et al.* (1998) described two autonomous P-elements inserted at the telomere of an X chromosome in *Drosophila melanogaster*, in telomeric-associated sequences (TASs). TASs are known to possess the properties of heterochromatin, which can silence transgenes that are inserted within them. Knight *et al.* (2015) mapped the breaker element in *Gc2* to a location proximal to a block of subtelomeric heterochromatin. The breaker element was therefore hypothesized to be a transposon similar to the TAS P-elements referred to by Ronsseray *et al*. In this case, it is likely that the protector would be located close to the breaker in the subtelomeric region. In summary, the location of the breaker element demonstrated by Knight *et al.* supports a transposon theory for gametocidal mechanisms.

*Ac*/*Ds* TEs may be another potential line of inquiry. During a hybrid dysgenic event, an *Activator* (*Ac*) (potentially synonymous to the breaker element in gametocidal genes) may become mobile, leading to non-autonomous *Dissociation* (*Ds*) elements to become activated within wheat. However, if the cell has established silencing of *Ds* elements previously (potentially via the protector in gametocidal genes), less damage is likely to occur. This may provide support for a transposon theory that aligns with the dual-mechanism model.

There are also epigenetic similarities between gametocidal action, hybrid dysgenesis and TE activation. Epigenetic changes are the main regulatory mechanism during gamete development (Burn *et al.*, 1993; Amado *et al.*, 1997), corresponding to TE activation (Slotkin *et al.*, 2009). Since gametocidal action does not occur in somatic cells, there is a suggestion that gametocidal action is regulated by DNA methylation. In line with the dual-mechanism model (Endo, 1990) and some parts of the restriction-modification model (Tsujimoto, 2005), the silencing of the breaker element may occur through methylation by the protector element. As previously described, de Las Heras *et al.* (2001) exposed the root tips of Chr. 4S^sh^ monosomic and disomic translocation lines to 5-azacytidine, a DNA hypomethylating agent. In the CS control, there was no chromosome fragmentation. However, fragmentation and breakages of chromosomes were observed in plants carrying the translocation. The evidence presented implied that methylation of the breaker element that inhibits gametocidal action occurred post-embryo formation (King and Laurie, 1993). Moreover, it supported the dual-mechanism theory, whereby the protector element methylates the breaker element to prevent damage to the cell’s own chromosomes.

Nonetheless, this evidence does not suitably explain how the breaker element still affects cells beyond its own following silencing methylation by the protector. Tsujimoto (2005) highlighted the similarities to restriction-modification systems in bacteria where restriction enzymes are produced that cleave a specific site in the genome, unless the sites are methylated by the modification enzymes. Though experimental evidence to support this is lacking, several publications indicate that gametocidal action may take place at specific sites on the chromosomes (de Las Heras *et al.*, 2001; Su *et al.*, 2013). Evidence of specific target sites for gametocidal action would broaden support for the restriction-modification theory. However, since TEs often insert into centromeric and heterochromatic regions, it may suggest further investigation into the hybrid dysgenesis theory.

If specific target sites were confirmed, it would be interesting to observe if variation in sites exists between different gametocidal genes. Identifying variation, or lack of it, may elucidate the origins of gametocidal genes, whether of a single or multiple independent evolutionary events, or even that this is a process so common to many species that huge variation exists. Endo (1982) considered interactions between gametocidal genes in different species, which contributed to his proposal in grouping them according to homoeology and strength (Endo, 1990). Due to comparatively little knowledge of gametocidal genes at the time of publication, it is difficult to determine the particular gametocidal loci Endo was working with. Nonetheless, differences were observed in addition lines with multiple species of gametocidal chromosomes. Replicating this experiment with modern methods may allow a more substantiated understanding of the interactions, or lack of them, between gametocidal genes, species and/or groups.

The most extensive epigenetic studies into the molecular mechanisms have been undertaken by Wang *et al.* (2017, 2018), whose use of modern techniques has informed a number of substantiated conclusions. Wang *et al.* (2017) conducted a study to locate the areas of unique methylation on the genome of a monosomic and disomic Chr. 3C^t^ addition line. Methylation was exhibited more frequently and in differing places between the addition lines and control (CS), supporting prior hypotheses relating methylation to gametocidal action (de Las Heras *et al.*, 2001; Su *et al.*, 2013).

Furthermore, it was ascertained that several methylated sites were homologous to three TEs, one of which was hypomethylated and the other two hypermethylated. Hypomethylation of LINE-1 TEs has been associated with chromosomal instability (Muñoz-Lopez and Garcia-Perez, 2010; Martínez *et al.*, 2012) and therefore Wang *et al.* suggested that the gametocidal gene activates specific transposons by reducing methylation, causing TE mobility to specific positions and inducing breakages. The exception to the breakages would be if the specific target site was methylated and therefore protected, such as the hypermethylated transposons that were found. In 2018, Wang *et al.* presented further evidence for the roles of methylation during gametocidal action, alongside the roles of micro- and silencing RNAs (Wang *et al.*, 2018).

While some hybrid dysgenesis events involving TEs and epigenetics may be used to understand the dual-mechanism model suggested by Endo (1990) and the restriction-modification system proposed by Tsujimoto (2005) and recently recapitulated by Said *et al.* (2024), recent improvements in knowledge have weakened the plausibility of these theories. While methylation as a means of protection against breaker elements has been discussed in both Tsujimoto (2005) and Said *et al.* (2024), little more has been analysed with regard to the developmental stages and demethylation, alongside associated ncRNAs. Additionally, hybrid dysgenesis via activated TEs was not mentioned at all.

It may be likely that, rather than being based on two separate elements, a breaker and protector (or restriction enzyme and modification enzyme, as per the theory of Tsujimoto (2005)), the breaker is the genetic element responsible for chromosomal fragmentation, whereas the protector is a normal function of the cell, designed to maintain genomic integrity during major developmental stages, such as gamete formation. In a gametocidal event, the innate protective mechanism, responsible for supressing expression of harmful elements or genes not necessary for the developmental stage, does not have the genetic information, such as siRNAs, to do so. Thus, the breaker element, whether that be a transposon or an ill-timed gene, is activated, leading to fatal downstream effects. Furthermore, the suppressor element can potentially be explained through this innate silencing mechanism. If plants with a suppressive ability have homology to the breaker element, siRNAs will be produced during gametogenesis, as normal, during the gamete developmental stages that can intercept and silence them. While investigating *Igc1*, a suppressive element to gametocidal Chr. 3C^t^, Tsujimoto and Tsunewaki (1985) noted the gametocidal chromosome had homology to the chromosome containing the suppressive element.

The dual-mechanism and restriction-modification models that have been proposed have streamlined thinking into the consideration of only two elements. However, the involvement of multiple TEs in gametocidal action could explain why some publications suggest the element may be polygenic. For example, in attempts to remove the gametocidal gene, Marais *et al.* (2010) and also Ragini *et al.* (2024) observed that fertility in progeny over generations continued to vary. Marais *et al.* (2010) considered that the gametocidal gene may be multiple elements, with more being removed during each successive backcross. Furthermore, Wang *et al.* (2017) found that areas of differential methylation in gametocidal lines were homologous to three TEs. Should TEs be the cause of gametocidal action, this may also suggest that multiple elements could be responsible. Additionally, Endo (1990) commented on an observation of a different mutant phenotype caused by removal of part of the *Ae. sharonensis* Chr. 4S^sh^ chromosome. One explanation for this is that gametocidal action is on account of multiple elements. These publications are limited in their suggestion of the polygenicity of gametocidal elements due to a focus on the applied aspect of gametocidal gene removal, but nonetheless offer enough value for future consideration.

While the similarities between hybrid dysgenesis as a result of TEs and gametocidal action are undeniable, it must be a path followed with caution. Future studies should consider TEs, siRNAs and other non-coding RNAs at available opportunities. However, the symptoms of gametocidal action are in many ways vague and can potentially be associated with other problems during gamete and embryo development. Furthermore, the gametocidal gene may even be a novel finding. It is therefore necessary to contemplate all theories when undertaking this study without employing bias.

## REMOVAL OF GAMETOCIDAL GENES FROM WHEAT–WILD RELATIVE INTROGRESSION LINES

**Fig. 2.**
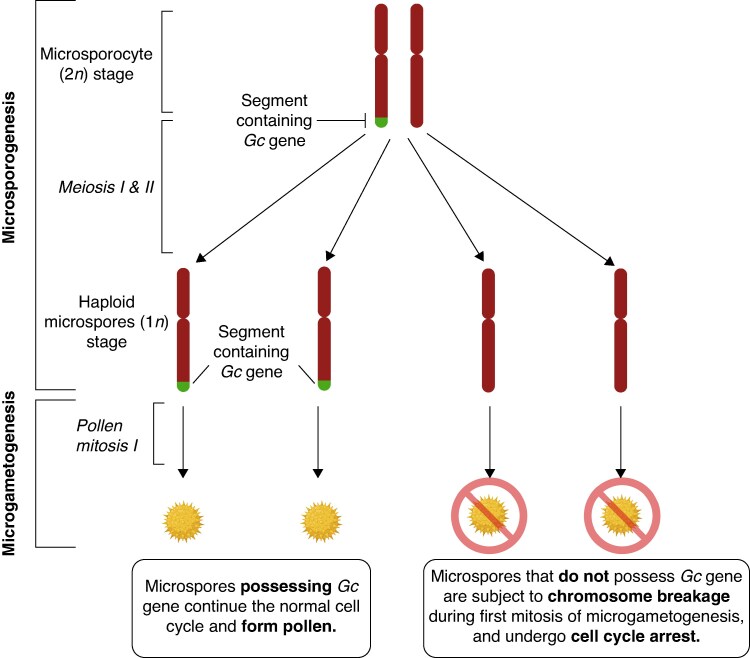
When gametes segregate in a hemizygous gametocidal plant, the gamete that does not contain the gametocidal element is subject to chromosomal breakage (Finch *et al.*, 1984; Nasuda *et al.*, 1998). This leads to a 50 % reduction in viable gametes and therefore a large yield penalty. Created in BioRender.

Much of the primary research on gametocidal genes has been in wild relatives of wheat. This is a result of the high value that wheat holds for global food security, and is a response to the challenges that gametocidal genes pose to breeding programmes. Within a breeding programme, intergeneric hybridization with wild relatives possessing gametocidal genes can become problematic (King *et al.*, 2018). For example, in a gametocidal line that is hemizygous for the element (i.e. *Gc*/*−*) (such as the offspring of wheat–*Ae. sharonensis* introgression line T4B–4S^sh^ (*Gc2*/*Gc2*) and wheat (−/−) parents) 50 % of the gametes are the subject of chromosomal aberrations during development (Fig. 2). This means that there is a significantly reduced seed set. In real terms, this is a huge reduction in yield and a line hosting a gene such as this, regardless of other useful traits, would not be tolerated for involvement in a breeding programme. Thus, methods to remove gametocidal genes from hybrid lines have been highly sought after.

There have been several attempts to disrupt the gametocidal gene using mutagens. Marais and Pretorius (1996) used *Ae. speltoides* as a source of genes for leaf rust and stem rust resistance but could not use the resulting offspring for commercial purposes due to the gametocidal locus (*Gc1*; Tsujimoto and Tsunewaki, 1985) from the alien species co-transferring alongside the desired gene. They treated the lines with either γ radiation or the mutagen *N*-nitroso-*N*-methylurea in an attempt to disrupt the gene. Some treated plants exhibited a higher rate of fertility and seed development, but this did not persist in subsequent progeny.

Marais *et al.* (2010) also attempted to separate and remove the gametocidal genes in another way, firstly by selecting for high fertility and secondly by attempting recombination of chromosomes in both the presence and absence of *Ph1.* Unfortunately, in both methods gametocidal action persisted in the progeny. However, the gametocidal action appeared to be reduced, and therefore Marais *et al.* suggested that this indicated that the gametocidal element may in fact be polygenic and some of the genes responsible had been successfully removed. It was suggested that further selection of highly fertile heads may remove more of the gametocidal elements, rendering the line suitable for breeding programmes.

Friebe *et al.* (2003) also successfully attempted to create a knockout gametocidal mutation line, through treatment of a wheat–*Ae. sharonensis* introgression line with EMS. They subsequently characterized the mutant line T4B–4S^sh^#1 (frequently referred to as Gc2mut), through test crosses to ensure the line had fully lost the phenotype of chromosomal breakage. Millet *et al.* (2017) then successfully removed the gametocidal effect by making use of this mutant line. After completing the selected crosses to introgress a gene from *Ae. sharonensis* associated with African stem rust resistance (i.e. Ug99 group races) into wheat, the individuals were crossed with the mutant *Gc2* line, T4B–4S^sh^#1, or Gc2mut. Since the *Gc2* mutation is dominant and overcomes the semi-sterile phenotype of the wild-type *Gc2* gene (Friebe *et al.*, 2003), the resulting progeny inherited the faulty gametocidal gene, ensuring high fertility in future generations. Homozygous *Gc2^mut^*/*Gc2^mut^* plants were identified that only carried the introgression from *Ae. sharonensis* responsible for the stem rust resistance. However, the extent to which the fertility was restored was not mentioned. As was suggested by (Marais *et al.*, 2010) when attempting to remove the gametocidal gene from *Ae. speltoides*, a less than full seed set may indicate that some gametocidal action may remain, suggesting that multiple gametocidal elements may be present. This could be an interesting point of comparison between the gametocidal gene of *Ae. speltoides* (likely to be *Gc1*; Endo, 2007) and *Ae. sharonensis Gc2* and the methods that were used.

While this method appeared successful in working around the *Ae. sharonensis* gametocidal action, it is not commercially ideal. Using the Gc2mut line at the last stage makes it a timely process and less efficient working with 50 % seed set for the prior generations. The technique is also currently limited to *Ae. sharonensis*, and not useful for gametocidal genes in other species until respective mutation lines are produced. Finally, the technique relies on breeders possessing Gc2mut seed, making the technique potentially inaccessible. The EMS technique of Friebe *et al.* (2003) could be utilized to develop more mutant lines within breeding programmes but involves a timely and labour-intensive process using non-targeted mutagenesis.

Ragini *et al.* (2024) used a combination of techniques similar to Marais and Pretorius (1996), Marais *et al.* (2010) and Millet *et al.* (2017) in an attempt to remove *Ae. speltoides*-derived gametocidal action. The authors used γ radiation to induce a knockout mutation in a wheat–*Ae. speltoides* introgression line and used these mutants to backcross to wheat to remove the gametocidal elements. While it was claimed that the gametocidal gene(s) were removed, fertility was not fully restored, as was also observed in Marais *et al.* (2010). Once again, though not suggested by the authors, this may indicate that multiple genes responsible for gametocidal action exist on the alien chromosome. However, it should be noted that lower fertility may also have been a result of residual background mutations from γ radiation in fertility-related genes.

Finally, a promising method may be in mapping gametocidal elements for downstream targeted mutagenesis. Thus far, no gametocidal element has been precisely mapped or identified, though work towards this has been ongoing (discussed below). By mapping the gametocidal gene in the wild relative parent, a targeted knockout mutation could be induced to create a line with a faulty breaker gene, a process that could be quickly and widely disseminated. The technique used to locate the breaker gene could be transferred to other species, accelerating the process of developing other mutant lines for use in breeding programmes.

## MAPPING OF GAMETOCIDAL GENES IN WHEAT WILD RELATIVES

Due to their impact on modern wheat breeding programmes, efforts have been made to precisely map some of the gametocidal genes. Traditional method of positional mapping through observing segregation and linkage patterns cannot be used as a result of the preferentially transmitting nature of gametocidal genes. Innovative approaches have thus far been used to identify and map the elusive genes in *Aegilops* spp.

### *Gc2*: the *Ae. sharonensis* gametocidal locus and its mapping

The *Ae. sharonensis* gametocidal gene, *Gc2*, has been a primary research focus due to its strong gametocidal action (Endo, 1990, 2007) and therefore clear phenotype and large reduction of yield in introgression lines. Additionally, *Ae. sharonensis* has been demonstrated to contain many useful genes (Marais *et al.*, 2006; Olivera *et al.*, 2007; Millet *et al.*, 2017; Wang *et al.*, 2021; Sharma *et al.*, 2024), but its use is hindered in breeding programmes due to its gametocidal action. There have been several fundamental publications on the *Ae. sharonensis* gametocidal locus demonstrating its action (Finch *et al.*, 1984; Friebe *et al.*, 2003; Knight *et al.*, 2015), leading to a moderate understanding of what to expect when working with the locus. Furthermore, there have been publications detailing gametocidal wheat–*Ae. sharonensis* introgression lines (King *et al.*, 1991; Nasuda *et al.*, 1998; Endo, 2007), of which one has been used to create an EMS-induced loss-of-function gametocidal mutation line (Friebe *et al.*, 2003).

The gametocidal Chr. 4S^sh^ from *Ae. sharonensis* is homoeologous to wheat group 4 chromosomes. However, it tends to introgress into Chr. 4B (Nasuda *et al.*, 1998) or Chr. 4D (Miller *et al.*, 1982; King *et al.*, 1991, 1996). Nasuda *et al.* (1998) described the translocation line T4B–4S^sh^ (originally created by T. R. Endo, but unpublished), whereby an *Ae. sharonensis* segment with the gametocidal locus was translocated onto the long arm of Chr. 4B in wheat cultivar CS. Using a cytogenetic study, chromosomal fragmentation was demonstrated in developing pollen gametes, specifically at the interphase prior to the first mitotic cell division of microgametogenesis. The action was particularly strong when compared with Chr. 2C^c^ of the *Ae. cylindrica* gametocidal locus. However, recent research has indicated that translocations into Chr. 4D are more common, due to the closer relationship of the S genome with the D subgenome than the B subgenome (Marcussen *et al.*, 2014; Avni *et al.*, 2022; Li *et al.*, 2022; Yang *et al.*, 2023). This was observed by King *et al.* (1991, 1996), who used two lines with translocations of *Ae. sharonensis* Chr. 4S^sh^ into Chr. 4D of wheat cultivar ‘Brigand’.

Friebe *et al.* (2003) demonstrated chromosomal breakages in developing pollen of hemizygous *Gc2*/− lines using microscopy and linked the effect to the absence of the Chr. 4S^sh^ translocation (carrying the *Gc2* gene) using FISH, accumulating further evidence that gametocidal action takes place in gametes lacking the *Gc2* gene. The study also provided further support for Endo’s dual-mechanism theory (1990). To do this, Friebe subjected thousands of seeds to EMS treatment to induce point mutations, one of which landed in the breaker element of the gametocidal locus. This mutant, T4B–4S^sh^#1, lost the ability to cause chromosomal breakages in gametes but retained the ability to protect the gametes containing the mutated locus against gametocidal action. The study indicated that there may be at least two tightly linked elements involved in the gametocidal action of *Gc2*. The T4B–4S^sh^#1 line has been used in both research and pre-breeding programmes (Millet *et al.*, 2017).

As noted previously, the preferential transmission of gametocidal genes means that traditional methods of positional mapping through observing segregation and linkage patterns cannot be used. However, through cytogenetic studies, both Endo (2007) and Friebe *et al.* (2003) demonstrated that the Chr. 4S^sh^ translocation was on the distal end of the long arm of Chr. 4B in wheat. Knight *et al.* (2015) built upon this work by attempting to map the breaker element (*GcB*) in the *Gc2* gametocidal locus using deletion lines and DNA markers. The exact position of the gene is yet to be located on Chr. 4S^sh^, but thus far Knight *et al.* have mapped it to a block of subtelomeric heterochromatin on the long arm of Chr. 4S^sh^.

The research by Knight *et al.* applied a strategy similar to that used to define the *Ph1* locus (Griffiths *et al.*, 2006). The polyploidal nature of the wheat genome meant that traditional mapping techniques could not be applied, and therefore γ radiation was used to induce deletions. Using the 4DS-4DL-4S^sh^L translocation lines (King *et al.*, 1996), Knight *et al.* (2015) induced deletions as well as translocations of varying sizes involving the Chr. 4S^sh^ segment. The *F*_1_ generation of the translocation line and wild-type wheat crosses was phenotyped for fertility, and cytologically analysed during first mitosis of pollen for chromosomal fragmentation to identify whether *GcB* was present or absent. Markers were developed for the Chr. 4S^sh^ region and used to define the size of the deletion or translocations and identify the region of *GcB*.

The choice of γ radiation to induce mutations contrasted with previous methods, which used EMS (Friebe *et al.*, 2003) and 5-azacytidine (de Las Heras *et al.*, 2001). However, γ radiation tends to induce larger deletions rather than point mutations or methylation removal. As only the approximate location of the region was known, larger deletions may have had more use in initially narrowing down the region. To continue the work of Knight *et al.* (2015) and map *GcB* precisely, point mutations may be more appropriate. EMS, a mutagen that induces point mutations, was successfully used by Friebe *et al.* (2003) to create a wheat–*Ae. sharonensis* line with a mutation within the *Gc2* breaker element. Though no further information on the location of the mutation was realized due to technological limitations, this publication provided proof of concept for the methodology surrounding the development of a *Gc2-*containing line with a point mutation causing it to cease gametocidal action.

As highlighted in Knight *et al.* (2015) and Griffiths *et al.* (2006), genotyping assays are vital in fine-mapping a region on a chromosome. Single-nucleotide polymorphism (SNP)-based markers are useful to distinguish between two individuals. Prior to the release of the whole-genome sequence of wheat (International Wheat Genome Sequencing Consortium (IWGSC) *et al.*, 2018*a*), SNPs between wheat and *Ae. sharonensis* were more challenging to develop. Knight *et al.* (2015) used a draft transcriptome assembly of *Ae. sharonensis* (Bouyioukos *et al.*, 2013) and generated SNPs against wheat using primers designed on syntenic chromosome regions of *Oryza sativa*, while Grewal *et al.* (2017) developed SNPs using synteny with *Brachypodium distachyon* to find the corresponding wheat homologues. This explains why fine-mapping of *GcB* was not possible for Knight *et al.* at the time of publication. Since the availability of the wheat genome reference sequence, more specific and extensive bioinformatic analyses have been possible, with SNP discovery far easier using alignment tools. In theory, this should make marker development for mapping *Gc2* more efficient.

An additional difficulty in marker development is the allopolyploid nature of wheat. Finding homoeologous SNPs that differentiate between heterozygous and homozygous alleles in a hybrid line can be problematic with multiple homoeologous subgenomes present. Knight *et al.* (2015) highlighted this, and (Grewal *et al.*, 2020*a*) described the method to create chromosome-specific Kompetitive allele-specific PCR (KASP) genotyping assays. KASP markers, based on SNPs, are comparatively time- and cost-effective for genotyping of introgression lines, but remain subject to the challenges of homoeologous SNPs. Nevertheless, the method by Grewal *et al.* (2020*b*) has proven to be successful in many publications since (Fellers *et al.*, 2020; Grewal *et al.*, 2020*b*, 2021; King *et al.*, 2022; Steed *et al.*, 2022; Coombes *et al.*, 2023).

### Mapping of Chr. 3C^t^ of Ae. *triuncialis*

While *Gc2* remains the most sought after gametocidal gene, the gametocidal Chr. 3C^t^ of *Ae. triuncialis* has been the subject of advanced genomic analysis, rendering it a top candidate for the first gametocidal gene to be mapped. As described previously, Wang *et al.* (2017, 2018) undertook extensive epigenetic studies a monosomic and disomic Chr. 3C^t^ addition line. Wang *et al.* (2017) identified areas of unique methylation that were more frequently exhibited on the lines with gametocidal chromosomes. Wang *et al.* proposed that sites exhibiting hypomethylation may be involved in the breaker action, whereas hypermethylated sites were protected, supporting the restriction-modification theory. With further comparison to specific TEs, compounded by further studies on the roles of micro- and silencing RNAs (Wang *et al.*, 2018), this evidence provides new leads for candidates for gametocidal genes. Replication of the experiments by Wang *et al.* (2017, 2018) using different gametocidal genes, such as the highly studied *Gc2* of *Ae. sharonensis*, alongside subsequent comparison of the methylated sites between species, could elucidate to evolutionary origins, strengths and actions, alongside interactions between species.

### Future work towards mapping gametocidal genes

Work towards mapping gametocidal genes has remained sporadic, with the latest research towards the gene’s discovery in *Ae. triuncialis* by Wang *et al.* (2018). Since then, technologies and bioinformatic tools alongside genomic resources have improved drastically, spinning a positive light for the future of gametocidal gene discovery.

As previously discussed, evidence suggests that methylation and/or TEs may be involved in gametocidal action (Wang *et al.*, 2017 , 2018). The scope of data acquired from sequencing technologies has advanced and may streamline investigation into these areas. While Wang *et al.* (2017) used a methylation-sensitive amplified polymorphism (MSAP) technique, Oxford Nanopore Technologies (ONT) and PacBio sequencing have improved the ease and accuracy of identifying epigenetic modifications. Moreover, with the average read length constantly increasing for both ONT and PacBio, identification of repetitive elements such as TEs is considerably more likely.

Sequencing technologies have not only improved but have become much more accessible and cost-effective. The National Human Genome Research Institute (NHGRI) has tracked costs associated with sequencing over a number of years, demonstrating that the cost is now well below what is predicted by Moore’s law (Wetterstrand, 2023). Moreover, bioinformatic pipelines have further improved the efficiency of acquiring sequencing data. Adhikari *et al.* (2022) and Coombes *et al.* (2023) demonstrated using skim-sequencing that exceptionally low-coverage sequencing data can be used to determined segments of alien introgressions in crop backgrounds. This reduces the cost of acquiring sequencing data and may be used to quickly validate gametocidal segment sizes in lines in some cases.

With the improvement of sequencing technologies alongside reduction in cost, access to genomic data has drastically improved in recent years. This has resulted in the release of numerous chromosome-scale genome assemblies not just of cultivated crops, but also of pangenomes and wild relatives of crops. With sequencing costs lower alongside assembly pipelines becoming easier to execute, assembling genomes is becoming more widely common on a laboratory-by-laboratory basis. For gametocidal gene mapping, access to genomic data will be critical. Once areas of interested are identified, inspection and annotation of the region will prove paramount in fine-mapping and understanding the mechanism. It is promising that both wheat (International Wheat Genome Sequencing Consortium (IWGSC) *et al.*, 2018*a*) and many *Aegilops* and *Triticum* species, including *Ae. sharonensis* and *Ae. speltoides*, have been assembled to chromosome scale and annotated (Avni *et al.*, 2022; Li *et al.*, 2022; Grewal *et al.*, 2024), fast-tracking investigation into genomic elements once regions are identified. An improvement on this could be the development of pangenomes for different accessions of these species.

With the availability of genome assemblies, transcriptome sequencing data can be analysed with considerably more accuracy. For discovery of genes, transcriptome assembly remains at the forefront of methodology: through identifying changes in transcripts, or comparison of gene expression, between different phenotypes, genes can be associated with certain traits or validated. However, using transcriptome analysis for gametocidal gene discovery remains problematic. It firstly presumes that gametocidal genes are indeed protein-coding, which, as described earlier, may not be the case. However, should this be the case, it would be highly challenging to undertake RNA sequencing at the time of gametocidal action. In *Aegilops* spp., gametocidal action has been identified to take place at different times of pollen development, which may add confusion to the correct identification of gene expression and therefore the time for RNA extraction. Moreover, the RNA extraction and sequencing on what may be half of the pollen in a single anther could pose a technically challenging, though not impossible, task. Specialized techniques such as single-cell transcriptome sequencing (Backhaus *et al.*, 2022) may further improve the ability to capture transcript information.

## MAPPING OF GAMETOCIDAL GENES IN *ORYZA* SPP.

*Oryza* spp. have been reported to contain several hybrid sterility (HS) loci. However, there has hitherto been little comparison with gametocidal genes despite similarities. Many HS loci in rice that are associated with reproductive isolation result in similar symptoms, including semi-sterility, aborted pollen grains and lower yields. However, gametocidal action has distinct characteristics, including selfish, preferential transmission of gametocidal genes, segregation disorder and the method of action. Some hybrid sterility loci in *Oryza* spp. may, by definition, also be characterized as gametocidal genes.

In *Aegilops* spp., gametocidal action is characterized by chromosomal fragmentation occurring in gametes that do not contain the gametocidal gene (Finch *et al.*, 1984; Friebe *et al.*, 2003). Gametes that do contain the gene confer a level of protection against chromosomal fragmentation (Friebe *et al.*, 2003). However, in *Oryza* spp. hybrid sterility loci, while chromosomal fragmentation appears not to occur, toxin–antidote systems are frequently referenced (Bravo Núñez *et al.*, 2018).

However, even within these groups, consideration for the selfish nature of gametocidal genes must be considered. Recent studies of hybrid sterility loci in *Oryza* have shown that these systems often extend beyond simple allelic incompatibility. Loci such as S5 and S7, which include ORF genes, operate via a toxin–antidote (killer–protector) mechanism: one allele (‘killer’) induces abortion of gametes or embryo sacs lacking the corresponding ‘protector’ allele, resulting in segregation distortion and preferential transmission of the protector allele in hybrid backgrounds (Chen *et al.*, 2008; Yang *et al.*, 2012; Yu *et al.*, 2016). Although this selfish drive is only observed in hybrids between divergent lineages, it resembles the transmission advantage conferred by gametocidal genes, while also maintaining reproductive isolation through gamete abortion or reduced fertility. Thus, these hybrid sterility loci combine features of both classic incompatibility and selfish element dynamics in interspecific or inter-subspecific hybrids. This aligns to a greater degree with classic hybrid sterility and reproductive isolation.

### *Sa* locus

There are a number of loci in *Oryza* spp. that meet the gametocidal assumptions that have predominantly been characterized in wheat wild relatives. *Sa* is a locus comprising two genes, *SaM* and *SaF*, with most indica cultivars containing *SaM*^+^ and *SaF*^+^ haplotypes, whereas all japonica cultivars contain the divergent *SaM*^−^ and *SaF*^−^ variations consistent with wild rice varieties (Long *et al.*, 2008).

In japonica–indica hybrids, *Sa* has a drive-like inheritance advantage in male gametes: *SaF*^+^ enforces a bias by killing pollen with *SaM*^−^, ensuring its own propagation (Long *et al.*, 2008), a hallmark of gametocidal action. It does this through a post-meiotic toxic protein interaction, differing from the mechanism of gametocidal genes in wheat wild relatives, but aligning with a toxin–antidote gametocidal theory. It was identified as the first HS complex locus consisting of two adjacent genes, *SaF* and *SaM*. *SaF* encodes an F-box protein and *SaM* encodes a small ubiquitin-like modifier E3 ligase. In *Sa* heterozygotes, interactions among proteins from three alleles (indica *SaF* and *SaM* and japonica *SaM*) cause selective abortion of pollen grains carrying the japonica *SaM* allele. The selective elimination of gametes and selfish propagation leading to distorted segregation occurring in the *Sa* locus is highly characteristic of gametocidal genes.

Long *et al.* (2008) used test crosses, pollen microscopy, phenotyping and genotyping to identify the dominance of the different *Sa* loci, demonstrating that in heterozygous plants almost all male gametophytes containing *Sa^j^* were aborted. This is strikingly similar to work conducted by Friebe *et al.* (2003) on *Ae. sharonensis Gc2*. Similarly to Knight *et al.* (2015) and Grewal *et al.* (2017), genetic markers were used to narrow down the locus eventually to a 10-kb region in the recombinant rice lines, and identify two candidate genes. The authors noted that during their initial analysis it became clear that this gametocidal action was a result of a multiple gene interaction, and thus the two candidate genes were taken forward for validation through sequence and structural comparison of proteins, map-based cloning and RT–PCR.

Xie *et al.* (2017*a*) used CRISPR Cas9 genome editing to independently knock out the *SaF* and *SaM* alleles of an indica rice line to create hybrid-compatible lines. The gene-edited neutral alleles enabled the lines to be used to make hybrids with no pollen sterility, with all pollen viability and agricultural traits left intact, overcoming the gametocidal effect.

### *S13*/*qHMS1* locus

The *S13* locus, also known as *qHMS1*, has been found to exhibit selfish, gametocidal behaviour in *Oryza sativa* and *O. longistaminata* hybrids. *S13^l^* of *O. longistaminata* discriminately eliminates pollens containing the allele *S13^s^* from *O. sativa* in heterozygotes. You *et al.* (2023), referencing the locus as *qHMS1*, demonstrated through cloning and mutagenic studies that the locus consisted of two genes: *HPT* (*Hybrid Pollen Toxin*) and *HPA* (*Hybrid Pollen Antidote*), encoding the toxic protein and antidote protein, respectively.

Furthermore, Myint *et al.* (2024) performed genetic analysis to map the gene, identifying a chromatin-remodelling factor involved in the gametocidal action. The complete gene in *O. longistaminata*, *OICHR* (noted as likely identical to *HPT*; You *et al.*, 2023), is responsible for the destruction of male gametes, demonstrated through knockout mutagenesis by Myint *et al.* (2024). The authors investigated the locus by matching phenotyping and genotyping data, narrowing the region to 40 kb containing four open reading frames. Two of these were homologous to *HPT* and *HPA* genes in the *qHMS1* region (You *et al.*, 2023). *OICHR*, of *O. longistaminata*, was identified to be the gene responsible for the ‘killer’ action, with the truncated version in *O. sativa* appearing not to confer protection on gametes.

### *qHMS7* locus

The *qHMS7* locus meets many of the key requirements for classification as a gametocidal gene. In *O. sativa*, two genes, *ORF2-D* and *ORF3-D*, exist as a toxin–antidote system (Yu *et al.*, 2018). During early pollen development, *ORF2* produces a toxic protein, which is neutralized by *ORF3*. The issue arises in hybrids between *O. sativa* and wild *O. meridionalis*. The ORF2 homologue in *O. meridionalis* is often non-functional and *ORF3* is absent, meaning there is no protection for pollen spores. This results in selective elimination of gametes without the protective gene, leading to segregation disorder and preferential transmission. Through map-based cloning, Yu *et al.* (2018) narrowed down the locus to a 31.6-kb region, where sequence analysis, qRT–PCR and mutagenesis confirmed the likelihood of two of the candidate genes.

### *pf12*/*RHS12*/*Se* locus

The *pf12* (Zhou *et al.*, 2023), *RHS12* (Wang *et al.*, 2023*a*) or *Se* (Wang *et al.*, 2023*b*) locus in *O. sativa* causes gametocidal-like, hybrid sterility in japonica–indica hybrids. Wang *et al.*, 2023*b* used genotypic and phenotypic analysis to map the locus, and validated the genes involved using mutagenesis after sequence analysis. However, Wang *et al.* (2023*a*) took this further, demonstrating a level of functional analysis. The authors suggested that *iORF3* produces the DUYAO toxin in sporophytes, causing pollen disruption. However, in gametes containing *ORF4*, the authors found that JIEYAO antidote protein is produced, which targets DUYAO for degradation, preventing pollen abortion in those gametes carrying the antidote gene. While this locus does not induce chromosomal fragmentation, it relates directly to gametocidal action in its ability to selfishly propagate through selective targeting of gametes that do not contain the gene. The locus was characterized by Wang *et al.* (2023*a*) using genetic mapping, gene expression, biochemical and cytological analysis, mutagenesis and test crosses.

### *S1* locus

The *S1* locus was first identified by Sano *et al.* (1979) as an HS locus in a cross between *O. sativa* and *O. glaberrima*, functioning as a ‘gamete eliminator’ to those carrying the *O. sativa* allele. *Oryza glaberrima* carries an *S1* locus, which includes three genes, *S1A4*, *S1TPR* and *S1A6*, that form a toxin–antidote system (Xie *et al.*, 2017*b*, 2019). However, the *O. sativa* counterpart locus only carries a shortened *S1TPR* gene, which negates its ability to protect itself. In hybrids, the tripartite *S1* gene system in *O. glaberrima* is expressed in sporophytic cells, triggering a sterility signal. The *S1TPR* gene, encoding a protein containing two trypsin-like peptidase domains and a ribosome biogenesis regulatory domain, then confers protection by neutralizing the signal (Xie *et al.*, 2017*b*, 2019). However, gametes containing the truncated *S1TPR* gene donated by *O. sativa* are not able to confer the protective capacity, leading to abortion of these gametes. Since only gametes, both male and female, containing the full-length *S1TPR* gene survive, there is selective elimination occurring and a distorted segregation bias of gametes, fitting the characteristics of a gametocidal gene system.

### Application of *Oryza* spp. HS loci characterization research to other gametocidal genes

While there was little reference to gametocidal action in the aforementioned HS locus research in *Oryza* spp., it is clear that there are significant similarities of distinct characteristics that have previously been identified in predominantly *Aegilops* spp. However, in many of these cases, research appears to be more active and at advanced stages in *Oryza* spp. HS loci, which could be directly applicable to the characterization of gametocidal genes in wheat wild relatives and other species.

Many initial stages of locus characterization remain similar. Test crosses, pollen microscopy, phenotyping and genotyping are used extensively in gametocidal gene identification in wheat wild relatives (Finch *et al.*, 1984; Nasuda *et al.*, 1998; Friebe *et al.*, 2003; Knight *et al.*, 2015; Grewal *et al.*, 2017) and HS loci in rice and wild relatives (Long *et al.*, 2008; Yu *et al.*, 2018; Wang *et al.*, 2023*b*). However, characterization of HS genes in *Oryza* spp. has advanced further, with map-based cloning, sequence analysis and structural comparison of proteins and mutagenic studies (Long *et al.*, 2008; Xie *et al.*, 2017*b*; Yu *et al.*, 2018; Wang *et al.*, 2023*a*; Myint *et al.*, 2024). This may be owing to the smaller genome sizes of rice (∼389 mbp; International Rice Genome Sequencing Project and Sasaki, 2005) and its wild relatives (Stein *et al.*, 2018). Genomics research in wheat and its wild relatives remains notoriously complex and expensive, due particularly to their polyploidy, adding additional challenges to an already elaborate phenomenon.

Moreover, the research supporting HS locus characterization may provide interesting perspectives on the mapping of gametocidal genes in *Aegilops* spp. Some HS loci are homologues, for example S13 in *O. sativa* and *O. longistaminata* (Myint *et al.*, 2024), rather than completely unique genes. Furthermore, some have been identified as polygenic. Evidence suggests that two genes are often responsible (Long *et al.*, 2008; Yu *et al.*, 2018; You *et al.*, 2023; Wang *et al.*, 2023*a*, *b*; Myint *et al.*, 2024) and in some cases three (Xie *et al.*, 2017*b*, 2019). This may demonstrate that techniques to identify gametocidal genes may not be as straightforward as typical gene characterization.

Recent molecular characterization has also suggested that many HS loci observed in rice could be orthologous (Li *et al.*, 2020). In the quest to characterize the *S13* locus, Myint *et al.* (2024) observed that two candidate genes were homologous to *HPT* and *HPA* genes in the *qHMS1* region (You *et al.*, 2023), which suggested them as candidates for validation studies. This may suggest a benefit to wheat wild relative research when one gametocidal gene is characterized: a comparison with gametocidal loci in other species may yield clues for further gene characterization.

## GAMETOCIDAL GENES AS A TOOL FOR BREEDING PROGRAMMES

Gametocidal genes have predominantly proved a challenge to crop breeders working with wild relatives. However, ‘cuckoo’ chromosomes may also present novel genetic manipulation tools that appeal beyond those who work on wild relatives of crops.

In practice, gametocidal genes may hold significant potential to improve methods in crop breeding and our knowledge of wild relatives. By improving our understanding of gametocidal genes and their mechanisms, it may be possible to utilize them as a tool within breeding programmes. The initial research for these novel uses has primarily been on Triticeae but could be explored in further genera.

### Inducing intergeneric chromosomal recombination

Wild relatives have been exploited in breeding programmes for decades as a novel source of genetic variation. Most notably was Sears (1972) and the transfer of leaf rust resistance from *Ae. umbellulata* to wheat, improving food security and saving billions of dollars in the USA. With global food security and climate change being ever increasing issues, exploiting the diversity of wild relatives to incorporate desirable traits into wheat is more important than ever. In particular, genes for disease and insect resistance and drought and salt tolerance are highly prioritized for improving cultivars (Reynolds and Braun, 2022). However, intergeneric recombination is problematic, and its inefficiency, as well as its time-consuming and laborious nature, has led to its decline (Laugerotte *et al.*, 2022), with only a few specialist groups possessing the ability to implement these programmes successfully. Recent improvements in technologies have enabled a new surge of research that may subsequently improve efficiencies within intergeneric breeding.

Despite improvements, there are still some barriers to introgression of alien chromosomes. Both wheat and wild relatives need to exhibit synteny amongst their chromosomes to allow recombination (King *et al.*, 2018). In cases where chromosomes are collinear, a system manipulating the *Ph1* gene (a gene that facilitates pairing of chromosomes, usually preventing wheat and wild relative chromosomes from recombining) has been developed to allow recombination between homoeologous chromosomes (Griffiths *et al.*, 2006; Martín *et al.*, 2017). However, the gene order of many wild relatives has become non-collinear to wheat, which makes this recombination almost impossible.

Gametocidal factors can potentially circumnavigate the issue of incompatible gene order between genera. By inducing chromosomal breaks through gametocidal action, translocations between wheat and wild relatives can occur (Endo *et al.*, 1994; Shi and Endo, 1999, 2000; Friebe *et al.*, 2000; Masoudi-Nejad *et al.*, 2002). After the initial research and use, there was a decline in their exploitation for this purpose due to the laboriousness and inefficiency of the process. Proposals have been made to identify the genes and enhance the process through improvement in modern technologies, particularly in *Ae. sharonensis* due to its strong ability to induce breaks in both gametes (Finch *et al.*, 1984) and early embryos (King and Laurie, 1993). This would open up an opportunity for greater exploitation of wild relatives as a source of genetic diversity.

### Preferential transmission of useful germplasm

Furthermore, stronger gametocidal factors have potential in ensuring preferential transmission to offspring (King *et al.*, 1991, 1992). King *et al.* (1991) created a translocation line using plants monosomic for wheat Chr. 4D and carrying the *Rht2* gene responsible for dwarfing, and Chr. 4S^sh^ (referred to as 4SL) of *Ae. sharonensis* containing the gametocidal factor. In the absence of a homologous chromosome, Chr. 4D and Chr. 4S^sh^ united to form a translocated chromosome containing the *Rht2* gene with the desired trait and the gametocidal factor that ensured preferential transmission of the desired chromosome to offspring. However, King *et al.* (1991) noted that previous cases of translocation have occasionally had detrimental effects on agronomic performance and are therefore not all desirable for use. Alongside a lack of modern technology to assist in this type of breeding, the technique once more declined in use. However, with recent improvements in the efficiency of detecting introgressions and the presence of useful genes, this technique of linking a desired trait with the gametocidal gene could once again be of great use. Mapping the appropriate gametocidal gene would be paramount in the utilization of gametocidal genes in this way.

Using the same concept as King *et al.* (1992), whereby a linked gene will be preferentially transmitted, eliminating the need for selection, Knight *et al.* (2015) have additionally suggested that desirable genes could be linked using genetic modification. This would involve the gametocidal locus together with a gene of interest being present in a vector, which is then inserted by transformation into wheat. While genetically modified crops are yet to be assimilated into agriculture in the UK, this may be viable in countries more accepting of the technology.

### Production of deletion lines and chromosomal mutations

Amongst the homoeologous groups of gametocidal chromosomes, the severity of chromosomal breakage and the resulting mutations or sterility varies. Nasuda *et al.* (1998) demonstrated this difference in male gametophytes between Chr. 2S of *Ae. speltoides* and Chr. 4S^sh^ of *Ae. sharonensis* gametocidal factors, alongside Chr. 2C^c^L of *Ae. cylindrica.* Chr. 2C^c^L produced less severe breakages than Chr. 2S and 4S^sh^. *Aegilops sharonensis* Chr. 4S^sh^ is known to cause complete sterility in gametes lacking the gametocidal gene, whereas other gametocidal chromosomes that cause less severe breakages can lead to mutations, translocations and deletions while still remaining viable (Endo, 2007).

Such weaker gametocidal factors have been used extensively to produce deletion lines and induce chromosomal mutations (Endo, 1988*b*, 2007, 2015; Endo and Gill, 1996). Deletion lines and chromosomal manipulation are widely used methods for gene mapping by observing the modified phenotype, which can be helpful to inform cloning of genes (Werner *et al.*, 1992; Gill *et al.*, 1993; Kota *et al.*, 1993; Hohmann *et al.*, 1994; Mickelson-Young *et al.*, 1995; Delaney *et al.*, 1995*a*, *b*). Endo and Gill (1996) used Chr. 2C^c^ of *Ae. cylindrica* and Chr. 3C^t^ of *Ae. triuncialis* translocation lines to produce 400+ deletion stocks, which were consequently useful in mapping the wheat genome prior to modern sequencing technologies (Gill *et al.*, 1996; Faris *et al.*, 2000; Sarma *et al.*, 2000; Weng *et al.*, 2000; Qi and Gill, 2001; Qi *et al.*, 2003; Sourdille *et al.*, 2004).

A recent further use of gametocidal genes has been in the creation of mutations and translocations within an individual plant as a source of novel genetic material. Chromosomal rearrangement can be used to provide breeders with a source of novel plant material that can be used to improve genetic variability in crops. Chromosomal rearrangements are a natural phenomenon that can lead to new phenotypes and speciation, a fundamental part of evolution (Levin, 2004). The process usually happens during segregation in meiosis of gamete formation. As rearrangements can often lead to sterility or fatality of gametes or weaker phenotypes, few survive natural selection and the timeframe of new genetic material arising is extremely slow. However, gametocidal factors can accelerate this process. Kwiatek *et al.* (2017) used a translocation line with the Chr. 4M^g^ gametocidal factor found on *Ae. geniculata* for large-scale manipulations of chromosomes in wheat. Through inducing androgenesis following gametocidal action, Kwiatek *et al.* cultured and obtained several double-haploid individuals with chromosomal rearrangements. Chr. 4M^g^ is known for its strong gametocidal action (Friebe *et al.*, 1999), which suggests a similar process may be possible with Chr. 4S^sh^ of *Ae. sharonensis*.

Chr. 4S^sh^ has also been shown to have an effect on embryos and endosperm, providing an additional use in breeding processes (King and Laurie, 1993). Through inducing chromosomal breakages at this stage, mutations, translocations and deletions can be made, thus generating novel genetic resources and deletion lines.

## CONCLUSIONS

To improve global food security in the face of climate change and rising populations, significant improvements to major crops are needed. Many lack the genetic diversity to deliver new and impactful improvements. Wild relatives of crops are a novel source of genetic diversity that can be exploited to bring valuable traits, such as stress tolerance and resistance to pests and diseases. However, gametocidal genes have the ability to significantly hinder this.

Gametocidal genes ensure their preferential transmission to offspring through inducing chromosomal breakages in gametes that lack them, resulting in agronomically unsuitable crop lines. It is currently unclear how this happens, though a dual-mechanism model suggests that there is both a breaker element, causing the chromosomal abnormalities, and a protector element, which protects gametes that contain the gene against the action. However, molecular research suggests there may be more to the mechanism than previously hypothesized. Attempts to remove these genes have been varied, and mapping has not yet been possible.

While gametocidal genes primarily hinder breeding programmes with wild relatives, opportunities for exploiting them as a breeding tool have been approached in the past. Recent advances in technologies may allow their efficient use in future programmes.
